# Level-dependent effects of predation stress on prey development, lifespan and reproduction in mites

**DOI:** 10.1007/s10522-022-09980-z

**Published:** 2022-07-25

**Authors:** Xiaoying Wei, Zhi-Qiang Zhang

**Affiliations:** 1 School of Biological Sciences, The University of Auckland, Auckland, New Zealand; 2 Manaaki Whenua – Landcare Research, 231 Morrin Road, St Johns, Auckland, 1072 New Zealand

**Keywords:** Non-consumptive effects, Predation risk, Development, Reproduction, Lifespan

## Abstract

In predator–prey interactions, non-consumptive effects of predators have been less studied than consumptive effects. However, non-consumptive effects may have significant influences on prey and can change different aspects of their life history such as development, reproduction and lifespan. The odour and other cues associated with a predator, without direct contact, could induce stress in prey, leading to phenotypic changes in life history traits. In this study, we investigate how mild and strong predator-induced stress could affect prey life history. The prey (*Tyrophagus putrescentiae*) was exposed, from hatching to death, to three different levels of predation stress from its predator (*Neoseiulus cucumeris*) (1, 3 or 5 predator adults in an adjacent cage separated by a mesh screen). Compared with the control, both males and females under predator-induced stress had longer developmental time and shorter lifespan when the level of predation stress increased, showing significant level-dependence. In addition, females had reduced fecundity under predation stress. Sex-specific response to predation stress was observed under a low level of predation stress: females had greater reduction in lifespan than males. Furthermore, the reduction in female lifespan was due more from the decrease in the post-oviposition period than the decrease in the oviposition period. Future studies applying even milder levels of predation press, such as exposure of prey to predator cues only during part of the prey lifespan, may provide additional insights.

## Introduction

In predator–prey interactions, non-consumptive effects of predators have been less studied than consumptive effects. However, non-consumptive effects of predators, such as predation risks, are known to cause changes in the ecology, behaviour and physiology of the prey (Stoks 2001; Peckarsky et al. 2002; Hawlena and Schmitz 2010; Skelhorn et al. 2011; Zanette et al. 2011; McCauley et al. 2011; Clinchy et al. 2013; Hermann and Thaler 2014; Gurr et al. 2017) including prey life history traits such as development and reproduction in mite prey-predator systems (e.g. Grostal and Dicke 1999; Škaloudová et al. 2007; Choh and Takabayashi 2010; Freinschlag and Schausberger 2016; Li and Zhang 2019; Wei and Zhang 2019). Studies on predation risks in the past mainly focused on the short-term effects on prey, such as development, behaviour and reproduction (e.g. Abrams and Rowe 1996; Peckarsky et al. 2002; Rocha et al. 2020; Oliveira and Moraes 2021; Saavedra et al. 2022; Majchrzak et al. 2022). Long-term effects, such as effects on lifespan, are not often attempted because it is rather time consuming to apply predation stress for the whole lifespan of many animal species, and it is also difficult to separate the effects of direct killing from the indirect non-consumptive effects in many predator–prey systems (Li and Zhang 2019). In this study we focus on the effects of predation stress on lifespan using a mite predator–prey system which allows relatively easy application of predation stress of different levels over a long period (Wei and Zhang 2019).

The limited number of studies on the effects of predation stress on prey aging and lifespan so far has shown contradicting patterns. For example, Pietrzak et al. (2015) showed in two water flea species only accelerated the rate of aging in *Diaphanosoma*, but an earlier onset of aging in *Daphnia* when they were exposed to olfactory cues of their predatory fish; however, another study showed previously that guppies from high-predation site had higher mortality rates with longer lifespans (Reznick et al. 2004). More recently, Auld et al. (2016) exposed freshwater snails to chemical cues from predatory crayfish but found no significant difference in the survival curves between treatment and control. Pietrzak et al. (2020) exposed two *Daphnia* species to their invertebrate predators but found no significant difference in age-specific survival between treatment and control. However, Pan et al. (2018) exposed *Brachionus* rotifers to predator-derived kairomones, which significantly decreased their lifespan. The causes for these inconsistent results are not well known and call for more detailed studies using different prey-predator systems.

Mites are useful but under-studied models for exploring the effects of predation stress on prey lifespan and aging. Earlier studies on mite behavioural responses to predator cues showed that prey avoided areas previously exposed to predators (Kriesch and Dicke 1997; Grostal and Dicke 1999). Later studies showed that prey males avoided to deposit spermatophores (Michalska 2016) and prey females avoided to lay eggs in areas previously exposed to predators (Walzer and Schausberger 2012). Predation stress was showed to prolong immature development (Freinschlag and Schausberger 2016), delay the onset of prey oviposition (Hackl and Schausberger 2014), reduce prey oviposition (Choh et al. 2010; Hackl and Schausberger 2014; Jacobsen et al. 2016); increase prey activity (Hackl and Schausberger 2014) and prey aggregation behaviour (Dittmann and Schausberger 2017). Recently, our group has been using different mite prey-predator systems to examine how predation stress affect prey lifespan. Li and Zhang (2019) hypothesised that prey under predator-induced stress should age faster with shorter lifespans and this effect should be sex-dependent: it should be more pronounced in females than males, which normally invest less than their female partners in reproduction and somatic maintenance. Using a mite prey-predatory system, Li and Zhang (2019) showed that spider mites (*Tetranychus urticae*) exposed to cues left on bean leaves by their predator (*Phytoseiulus persimilis*) showed prolonged development regardless of sex, but only female prey had shortened lifespan, as well as reduced lifetime fecundity. In this study, the exposure of leaves to predatory mites and the daily replacement of such predator-exposed units for the whole prey lifespan during the experiment was very time-consuming. Thus, Li and Zhang (2019) only studied one level of predation-induced pressure: predator cues deposited by one adult female predator in 24 h. Li and Zhang (2019) focused on transgenerational and sex-specific effects of predation risks in their study, which was not designed to examine the effects of different levels of predation risks on prey fitness. In this study, we extended the work of Li and Zhang (2019) by exploring level-dependent effects of predation stress on prey fitness using another mite predator–prey system (*Neoseiulus cucumeris* and *Tyrophagus putrescentiae*).

*Tyrophagus putrescentiae* was chosen as the experimental species in this study, because it is a common species that is widespread on plants and in stored food and products (Fan and Zhang 2007), it has a relatively short life cycle, and its laboratory colony is easy to establish and inexpensive to maintain for experimental research. *Neoseiulus cucumeris* is a common predator of *T*. *putrescentiae* (Lee et al. 2020) and was used as the predator to exert predation stress in this study (Wei and Zhang 2019). A previous study already showed in a two-choice test that *T*. *putrescentiae* preferred the cell not previously exposed to its predator *N*. *cucumeris*, which indicates that *T*. *putrescentiae* could respond to the cues deposited by its predator *N*. *cucumeris* (Wei and Zhang 2018). Later, Wei and Zhang (2019) designed a modified setup consisting of two Munger cells separated by a screen for *T*. *putrescentiae* and *N*. *cucumeris* respectively. This design enabled easier application of long-term predation stress as well as changing the levels of predation stress. By using this new design, we examined the level-dependent effects of predation stress on prey fitness by varying the levels of predation stress simply by adding different numbers (1, 3, and 5) of predators in the predator cell. We tested the hypothesis that prey lifespan might be shortened under strong predator-induced stress but very mild levels of predation may have no effect on prey lifespan or even increase it (similar to the effects of immunization). We also tested the hypothesis by Li and Zhang (2019) that the effects of predation stress on prey lifespan and aging should be sex-specific (e.g. more pronounced effects in females than in males).

## Methods and material

### Mite colonies

The colonies of both mite species were purchased from Bioforce Limited in South Auckland, New Zealand. The colony of *T*. *putrescentiae* was kept on a piece of black plastic sheet (about 15 cm in diameter) on a water-soaked sponge that was surrounded by wet paper tissue. They were placed in a plastic pan (22 × 22 × 3.5 cm) with water. Both sponge and the paper tissue were saturated with water to prevent the mites from escaping. The dry yeast (*Saccharomyces cerevisiae*, a product by Goodman Fielder Limited, New Zealand) was used to feed the storage mites (see Li and Zhang 2016 for details). The colony of *N*. *cucumeris* was kept in a transparent 1000 ml plastic box (15 × 10 × 7 cm), and the box was surrounded with water kept in the plastic containers (35.5 cm × 23.5 × 12.0 cm), as well. They were fed with *T*. *putrescentiae*. The colonies of both species were kept in a temperature-controlled room at 25±2 °C and 16:8 (L: D). A humidifier was used to keep the relative humidity at 80±2%. All experiments were conducted under the same conditions.

### Preparation of cohorts of mites for experiments

#### Prey

A larger cell (15 mm diameter) in a plexiglass slide (40 mm wide, 40 mm long, and 3 mm thick) was prepared and applied with yeast and 20 eggs of *T*. *putrescentiae* from the colony. Five such larger cells were made to collect the 50–60 females of the same age, which were placed into several new larger cells and maintained for 24 h. The eggs laid during this period (i.e. 24 h) were then collected and used in the experiments.

#### Predators

The active adult females of *N*. *cucumeris* were collected from the colony and put into the bottom Munger cell.

### Experimental procedures

#### Control group without predator-induced stress

A modified Munger cell introduced by Wei and Zhang (2019) was used. Briefly, a sheet of plexiglass (25 mm wide, 38 mm long and 3 mm thick) with a cone shape (top diameter 9 mm, bottom diameter 7 mm) in the middle was used as the main part of the container. A mesh material (500 grids per square inch) was attached to the bottom of the Munger cell by using non-toxic glue (Think Creative, PVA Glue 500 g). Another sheet of plexiglass of the same size without the hole was used as the lid of the cell. They were clamped together to enable a cell for *T*. *putrescentiae*. A small droplet of yeast was made by mixing dry yeast and water and applied to the Munger cell by a fine-hair brush (size 000). After *T*. *putrescentiae* became adults, they were paired in a Munger cell. The whole lifespan of *T*. *putrescentiae* was recorded by checking each cell daily. There were 70 replicates for the control group.

#### Treatment groups with predator odour

The containers for keeping *T*. *putrescentiae* were the same as the control group. However, there was a neighbouring cell screened by a mesh material against the bottom of the original container and the other side of the neighbouring cell was covered with the mesh material. This neighbouring cell was used to contain the predatory mite *N*. *cucumeris*. The eggs of *T*. *putrescentiae* used to feed the predatory mite were killed in a freezer (− 18 ℃) for 24 h. The dead prey eggs were then put into this neighbour cell to feed the predator mites. Thus, the odour of the predators could transmit through the mesh material, whereas the predator and the testing prey could not contact each other directly. Fresh predatory mites along with frozen eggs of prey were added to the bottom cell every 5–7 days. Three treatment groups of different levels of predator-induced stress were included: (1) LPS: Low predator-stress group: one predatory mite was placed in the neighbouring cell. (2) MPS: Medium predator stress: The same as (1) but with three predatory mites. (3) HPS: High predator-stress: the same as (1) but with five predatory mites. The predatory mites used were all young adult females of the same ages. There were 70 replicates for each treatment and observations of developmental status and daily reproduction were made until all *T*. *putrescentiae* died; they were then mounted individually on a glass slide using Hoyer’s medium, flattened by pressing the cover slip, and dried in an oven heated at 50 °C for at least 2 weeks before measuring body size (represented by the length of the prodorsal plate) under a Nikon optical microscope (Li and Zhang 2018).

### Statistics

R (version 3.6.0) was used to analyse experimental data. Because both the original data and the log-transformed data of immature developmental time were not normally distributed, a non-parametric test (Scheirer-Ray-Hare test) was used to test how the two factors (sex and treatment) influenced the different experimental groups and whether there were interactions between these two factors. Subsequently, the Kruskal–Wallis rank test was used to test the influence of the single factor when there was no interaction. The Wilcoxon signed-rank test was used for pairwise comparisons between treatments.

A Cox proportional-hazards model was used to compare survival across treatments and sexes. Both predictor variables and their interactions were analysed. The Kaplan–Meier survival analysis with log-rank test was used to make pairwise comparisons between treatment groups.

The different female reproductive parameters, including pre-oviposition period, oviposition period, post-oviposition period and fecundity (total number of eggs per female), either did not conform with normal distribution or to homogeneity of variance, or both. Thus, Kruskal–Wallis rank tests were used to test these parameters, and pairwise comparisons were analysed by the Wilcoxon signed-rank test.

Body size data were not normally distributed and did not display homogeneity of variance, so a Scheirer-Ray-Hare test was used to examine the effects of body size by sex and treatment as well as the interactions between sex and treatment. This was followed by the Kruskal–Wallis rank test to test the effects generated by these two factors respectively. The Wilcoxon signed-rank test was used for pairwise comparisons.

Spearman’s rank correlation was used to test the relationship between fecundity and lifespan; and between body size and lifespan, because they were not normally distributed.

All data are available to qualified researchers for meta-analysis upon requests to authors.

## Results

### Immature development and survival

Among the 70 replicates per treatment group, most individuals successfully developed to adults: 69 (37 females and 32 males) individuals in control group, 65 (32 females and 33 males) in the LPS group (low predation stress from 1 predator), 64 (32 females and 32 males) in the MPS group (medium predation stress from 3 predators), and 58 (29 females and 29 males) in the HPS group (high predation stress from 5 predators). Although the survival rates of these groups showed a decreasing trend with increasing levels of predator-induced stress, there was no significant difference in immature survival rates among treatments (*χ*^2^ = 0.01, df = 3, *P* = 0.997). For this experiment, the egg hatching rates of different groups were the same as the survival rates (all larvae and nymphs successfully developed to the next stage).

**Fig. 1:**
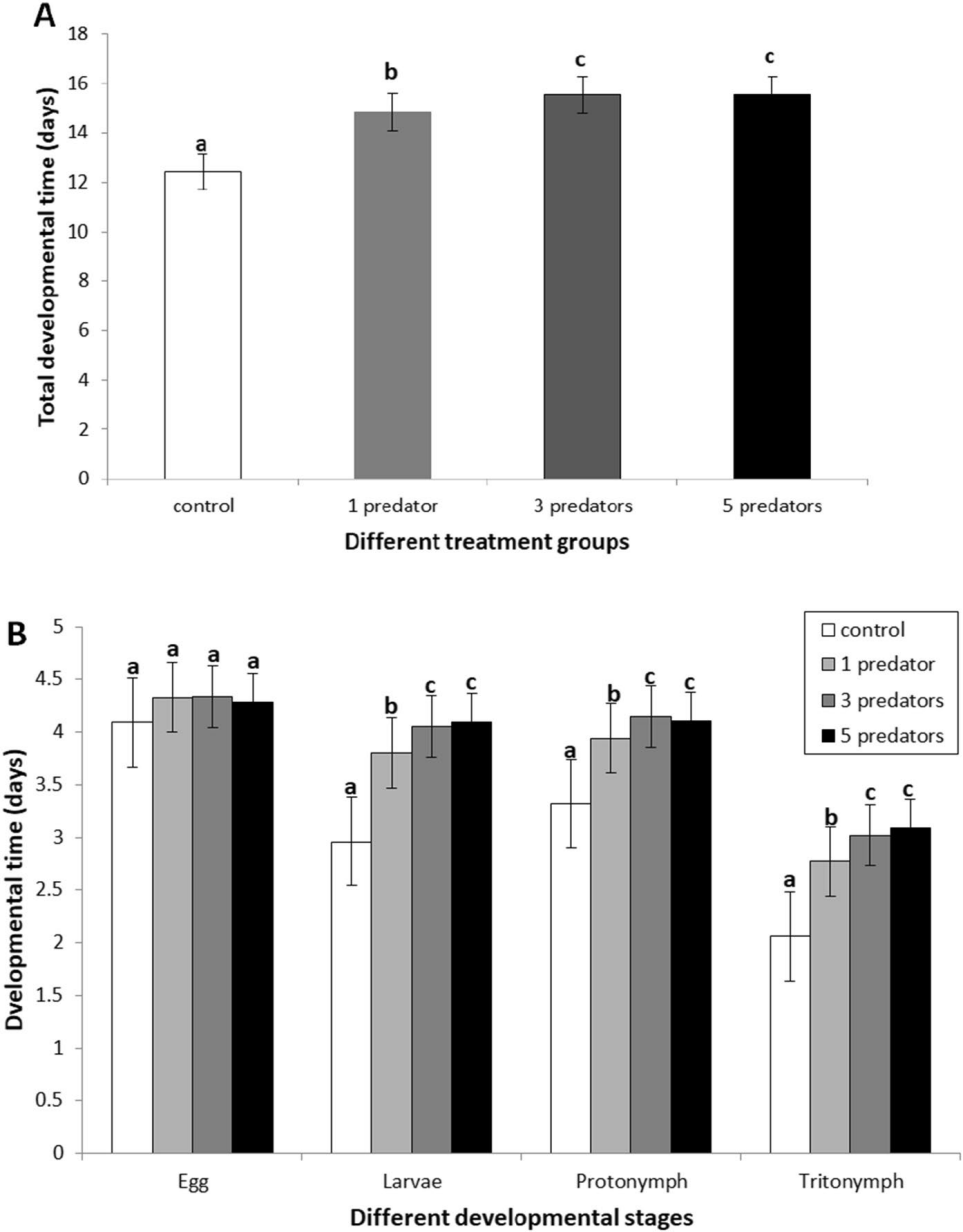
Developmental time of *T*. *putrescentiae* under different levels of predation stress (exposure to cues from 1, 3 or 5 predators, *N*. *cucumeris*). **A** Total developmental time of *T*. *putrescentiae* under different levels of predation stress. **B** Duration of each developmental stages of *T*. *putrescentiae* under different levels of predation stress. Note: Bars with the same letters are not statistically different at *P* = 0.05

Males reached maturity slightly faster than females in all the groups, although sex had no significant effect on developmental time (df = 1, H = 0.132, *P* = 0.716, Table 1) and was no interaction with treatment (df = 3, H = 0.277, *P* = 0.964). However, the main treatment effect was highly significant: the total developmental time was longer with increasing level of predator-induced stress (df = 3, H = 195.48, *P* < 0.001; Fig. 1A). To be more specific, the total developmental periods of the treatment groups were significantly longer than that of the control group (all *P* < 0.001), and the total developmental times of the MPS group and HPS group were longer than that of the LPS group (all *P* < 0.001). However, the difference between MPS and HPS groups was not significant (*P* = 0.879).

**Table 1:** Total developmental time of *T*. *putrescentiae* females and males under different levels of predatory stress

Total developmental time (days)	Control	Different levels of predation stress exerted by			χ^2^	*df*	*P*
		1 predator	3 Predators	5 Predators			
Females	12.42 ± 0.59a	14.84 ± 0.41b	15.54 ± 0.50c	15.55 ± 0.50c	92.48	3	< 0.001
Males	12.42 ± 0.50a	14.83 ± 0.42b	15.53 ± 0.50c	15.55 ± 0.50c	102.07	3	< 0.001

Means (± se) in the same row with different letters are statistically different at *P* = 0.05

A similar trend was found in different immature stages, except in the egg period, which showed no difference among treatments (*P* = 0.861; Fig. 1B). Individuals in all three treatments spent longer time than the control in three immature stages (larvae, protonymph, and tritonymph) (all *P* < 0.001). The MPS group took longer time than the LPS group in the three immature stages (*P* = 0.001 for larvae, *P* = 0.004 for protonymph, and *P* = 0.031for tritonymph) and HPS group also took longer time than the LPS group (*P* = 0.004, *P* = 0.015, and *P* = 0.004). However, the MPS group and HPS group were not significantly different in duration for each developmental stage (*P* = 0.255, *P* = 0.67, and *P* = 0.404).

### Reproduction, lifespan, and body size

All five reproductive parameters of *T*. *putrescentiae* were significantly influenced by treatment: pre-oviposition period increased (i.e. the onset of reproduction delayed) whereas the other parameters decreased with the increasing level of predation-induced stress (Table 2). Specifically, LPS did not delay the onset of oviposition of *T*. *putrescentiae* (*P* = 0.357), MPS delayed it by 28% (*P* = 0.032) and HPS by 65% (*P* < 0.001) (Table 2); the difference in pre-oviposition between LPS and MPS treatments was not significant (*P* = 0.195).

**Table 2:** Reproductive parameters of *T*. *putrescentiae* females under different levels of predation stress

Parameters	Control	Different levels of predation stress exerted by			*P*
		1 Predator	3 Predators	5 Predators	
Pre-oviposition period (days)	1.32 ± 0.09a	1.44 ± 0.10ab	1.69 ± 0.10b	2.18 ± 0.14c	*P* < 0.001
Oviposition period (days)	26.29 ± 0.62a	26.37 ± 0.46a	25.21 ± 0.33a	19.25 ± 0.60b	*P* < 0.001
Post-oviposition period (days)	39.89 ± 2.59a	23.63 ± 1.17b	26.10 ± 2.22b	19.29 ± 0.50c	*P* < 0.001
Daily reproductive rate (eggs/female/day)	16.17 ± 0.27a	7.32 ± 0.21b	5.85 ± 0.18c	6.50 ± 0.27c	*P* < 0.001
Lifetime fecundity (eggs/female)	423.04 + 9.55a	192.52 ± 5.97b	147.79 ± 5.30c	124.32 ± 5.80d	*P* < 0.001

Means (± se) in the same row with different letters are statistically different at *P* = 0.05

Neither LPS nor MPS significantly shortened the oviposition period, but HPS shortened it by 27% (Table 2). HPS shortened the post-oviposition period by 52%, whereas LPS and MPS shortened it by 41% and 36%, respectively (Table 2); the latter two were not significantly different from each other (*P* = 0.396).

LPS reduced the rate of oviposition by 55%, MPS by 64%, and HPS by 60%; the latter two were not significantly different from each other (Table 2). LPS significantly reduced the fecundity of *T*. *putrescentiae* by 54%, MPS by 64% and HPS by 71%.

**Fig. 2:**
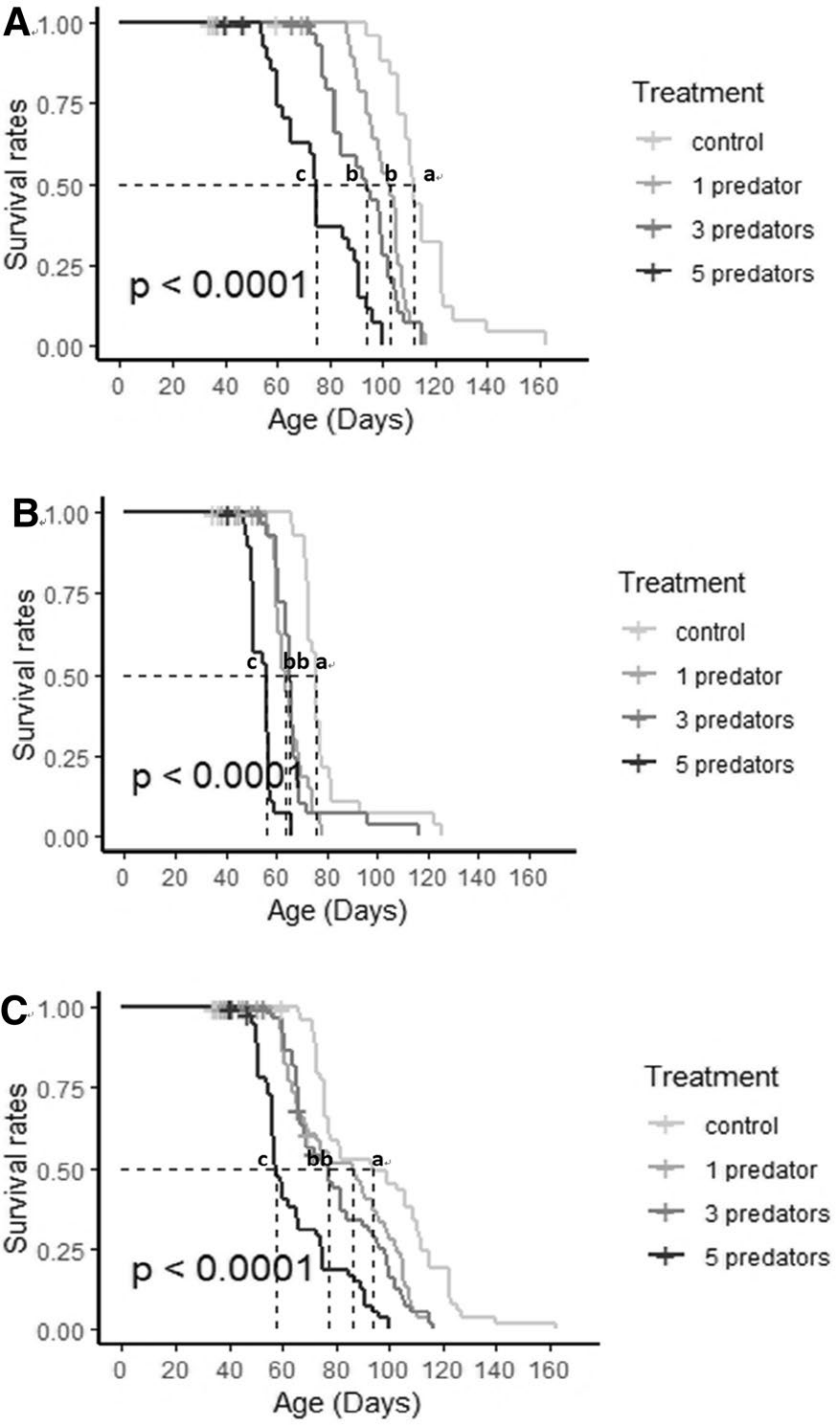
Survival rates of *T*. *putrescentiae* under different levels of predation stress **A** Survival rates of males of *T*. *putrescentiae* under different levels of predation stress. **B** Survival rates of females of *T*. *putrescentiae* under different levels of predation stress. **C** Survival rates of *T*. *putrescentiae* with male and female pooled together under different levels of predation stress. Note: The treatment numbers 0, 1, 3, and 5 refers to control, predation stress from 1 predator, predation stress 3 predators, and predation stress 5 predators, respectively. Lines with the same letters are not statistically different at *P* = 0.05

Comparison of the survival curves revealed that the two sexes had similar trends in survival rates (males: *χ*^2^ = 93.8, df = 3, *P* < 0.001 and females: *χ*^2^ = 122, df = 3, *P* < 0.001) (Fig. 2A, B). Besides, there were significant treatment effects (*χ*^2^ = 82.5, df = 3, *P* < 0.001) and the lowest survival rates were observed in individuals under HPS (Fig. 2C). Specifically, except for individuals of LPS and MPS groups, which had similar survival rates (*χ*^2^ = 0.7, df = 1, *P* = 0.4), all other pairwise comparisons were significant (all *P* < 0.001) (Fig. 2C). Comparing the sexes separately, similar patterns were found in both males (LPS-MPS: *χ*^2^ = 3.9, df = 1, *P* = 0.05) and females (LPS-MPS: *χ*^2^ = 0.2, df = 1, *P* = 0.7).

**Fig. 3:**
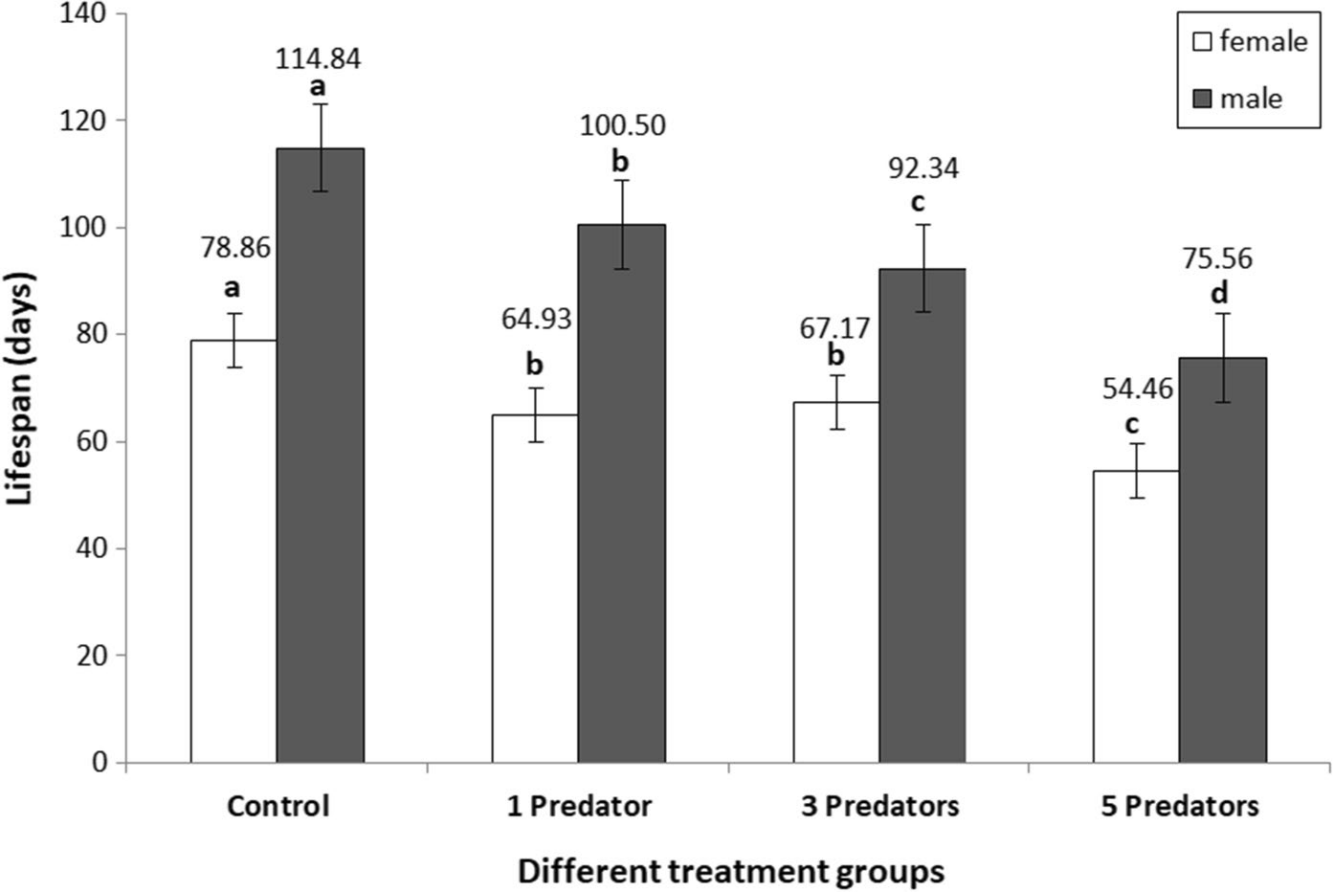
Lifespan of males and females of *T*. *putrescentiae* under different levels of predation stress. Note: Points with different letters are statistically different at *P* = 0.05

Moreover, we also found that with the increasing level of predation stress, prey individuals had reduced lifespan (H = 65.014, df = 3, *P* < 0.001), and in each treatment group, males consistently lived longer than females (H = 101.524, df = 1, *P* < 0.001) (Fig. 3). However, there was no difference in the trends between males and females under different stress levels (H = 1.829, df = 3, *P* = 0.608) (Fig. 3). Both males (*χ*^2^ = 62.281, df = 3, *P* < 0.001) and females (*χ*^2^ = 74.217, df = 3, *P* < 0.001) responded significantly to treatment, and mites under HPS lived shortest. However, there was a significant difference in the comparisons of LPS and MPS groups between males and females: in males, prey individuals of MPS group lived for a shorter period than those of LPS group (*P* = 0.009), while no such difference was seen in females (*P* = 0.5).

**Fig. 4:**
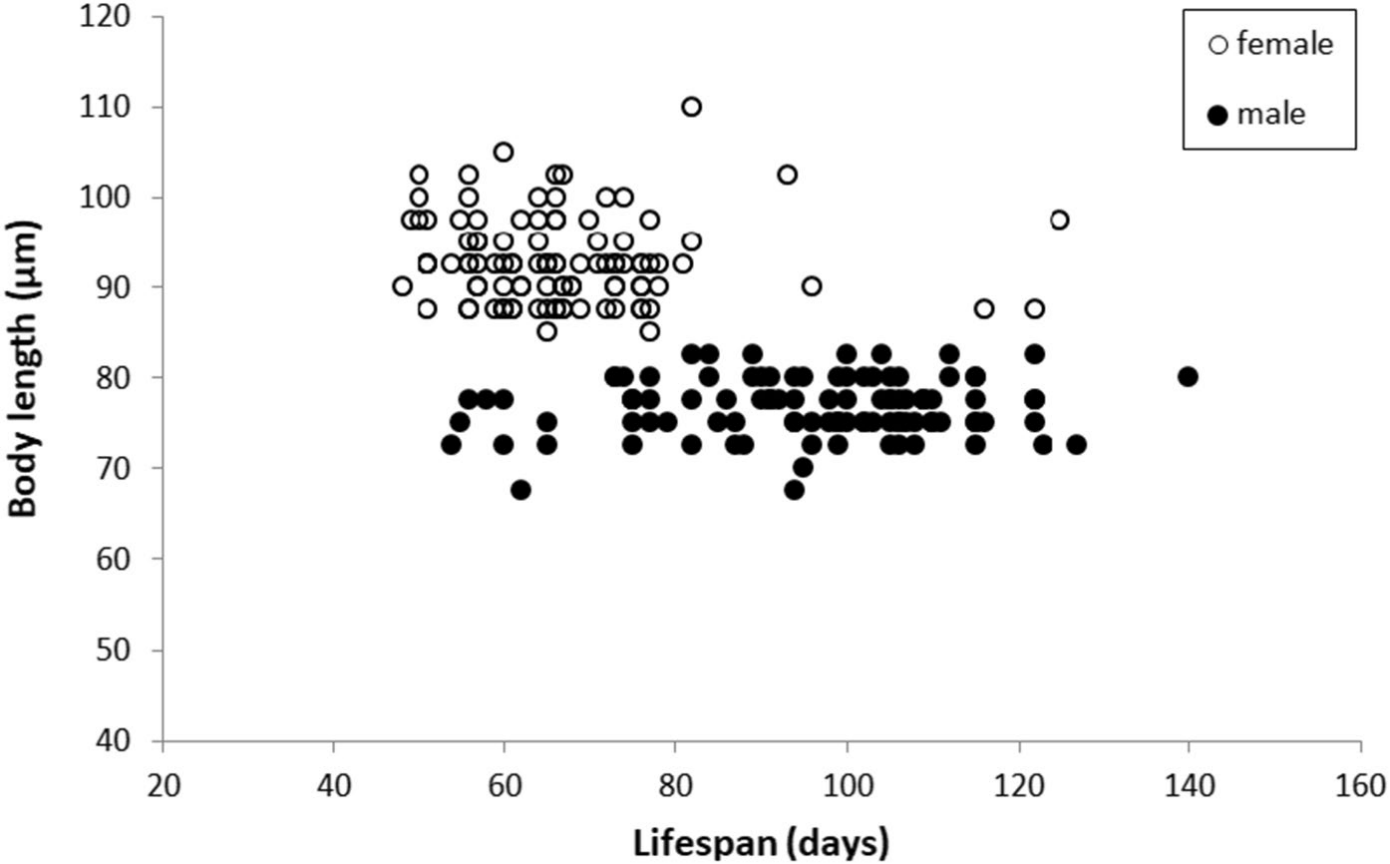
Body size (prodorsal shield length) of *T*. *putrescentiae* in relation to lifespan for males (solid dots) and females (open circle)

**Fig. 5:**
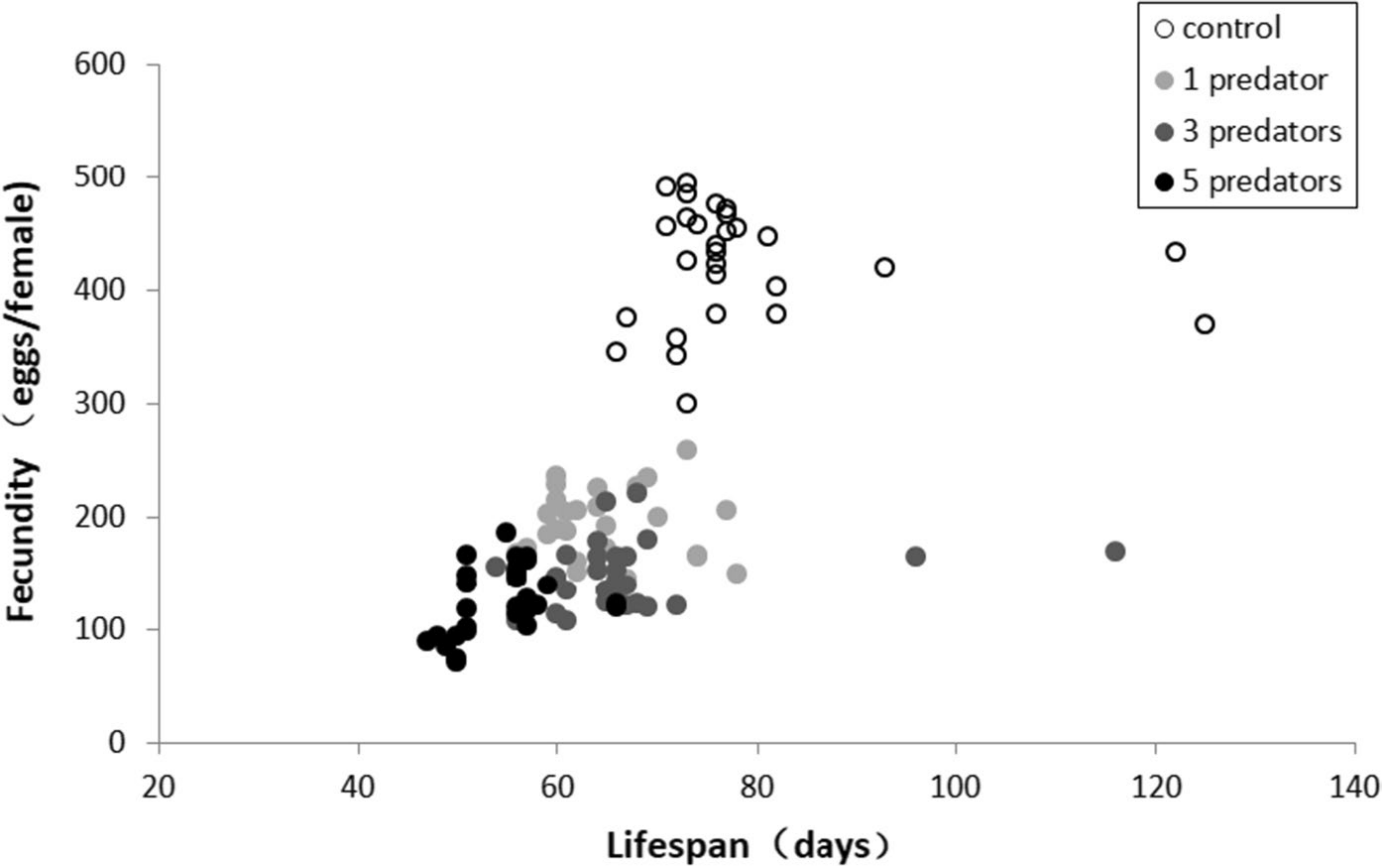
Fecundity of *T*. *putrescentiae* in relation to lifespan for females

Although females were significantly larger in size than males (*P* < 0.001; Table 3), the treatments did not have an influence on the body size of individual mites (*P* = 0.867) and did not interact with the sex (*P* = 0.666). Analysed separately for the sexes, treatments did not affect the body size in either males (*P* = 0.433) or females (*P* = *0*.083). There was no correlation between lifespan and body size (Fig. 4) in either males (*P* = 0.817) or females (*P* = 0.196). When the data for males and females were pooled, the body size was inversely correlated with the lifespan (*P* < 0.001, rho = − 0.597), reflecting that smaller males lived long than larger females (Fig. 4). We also found that across all treatments, fecundity was positively correlated with the lifespan (rho = 0.676, *P* < 0.001; Fig. 5).

**Table 3:** Body length (prodorsal shield length) of *T*. *putrescentiae* under different levels of predation stress exerted by 1, 3 or 5 predator females (*N*. *cucumeris*)

Body length (μm)	Control	Different levels of predation stress exerted by			χ^2^	*df*	*P*
		1 Predator	3 Predators	5 Predators			
Females	93.30 ± 5.45	91.76 ± 4.59	91.92 ± 4.65	94.46 ± 4.39	6.682	3	0.083
Males	76.98 ± 2.85	76.16 + 3.37	77.32 + 3.33	75.94 ± 3.02	2.744	3	0.433

Data in the format of means ± se

## Discussions

Non-consumptive effects—such as predation stress—on prey fitness have been demonstrated in previous studies on different prey-predator systems (Abrams and Rowe 1996; Kroon et al. 2004; Creel et al. 2009; Hawlena and Schmitz 2010; Gurr et al. 2017). Most of these studies concern the influences of predation stress on prey development or reproduction in relatively short-term experiments (Warkentin 1995; Walzer and Schausberger 2009; Choh et al. 2010; Janssen et al. 2014; Jacobsen et al. 2016; Rocha et al. 2020; Oliveira and Moraes 2021; Saavedra et al. 2022), because it is more difficult to conduct long-term experiments with larger animals that have relatively long lifespans while applying predation stress over an extended period of time (Li and Zhang 2019). In addition, it is also not easy to experimentally separate the effects of direct killing from the indirect non-consumptive effects in long-term studies. Using a mite prey-predator system in which both predators and prey have shorter lifespans compared with large animals, Li and Zhang (2019) overcame such technical difficulties and showed that predation stress extended developmental time of both females and males, and reduced fecundity and lifespan of the female. However, Li and Zhang (2019) studied only one level of predation-induced pressure in that study: i.e. predator cues deposited by one adult female predator in 24 h, because it is very time-consuming to expose leaves to predatory mites every day and to replace such predator-exposed units every day. Employing a different mite prey-predator system with the design of a set of two detachable cages separated by mesh screens, Wei and Zhang (2019) established a prey-predator system of *T*. *putrescentiae* and *N*. *cucumeris* that allowed the prey to be exposed to cues from the predator without being killed. This system makes the application of constant predator-induced stress easier. Wei and Zhang (2019) only examined the effects of predation stress on prey development and reproduction, as it was a short-term study to test a new method and new rearing cage. In this study, we used this new system (Wei and Zhang 2019) to test three different levels of predation stress on all life-long fitness parameters of the prey. We showed experimentally for the first time in a terrestrial animal the level-dependent effects of predation stress on prey lifespan and other fitness traits. We found that prey exposed to predation stress had longer developmental time, lower fecundity and shorter lifespan, and the effects were broadly level-dependent (i.e. stronger effects at higher levels of predation stress; Figs. 1 and 2; Table 2). The only other study of level-dependent effects of predation stress on prey lifespan involved aquatic rotifer prey and predators (Pan et al. 2018): *Brachionus* rotifers were exposed to three kairomone levels of *Asplanchna* predators, which reduced prey lifespan in an inverse level-dependent manner when food level was high, but similar level-dependence was not observed when food level was low.

A few previous studies on the effects of predation stress on prey aging and lifespan so far have shown contradicting patterns (Pietrzak et al. 2015, 2022; Auld et al. 2016; Pan et al. 2018; Li and Zhang 2019). The reasons for these inconsistent results are not well known. One reason could be the level-dependence of effects of predation risks on prey aging and lifespan. For example, *Brachionus* rotifers exposed to 40 ind L^−1^ of *Asplanchna* predators had the same lifespan as those in a control but had increasingly shorter lifespan at higher densities of predator kairomones of 80 and 120 ind L^−1^ when food level was high (Pan et al. 2018). However, in this study on *T*. *putrescentiae* and *N*. *cucumeris*, LPS (low predation stress from 1 predator) could decrease prey survival rate and reduce prey lifespan, although we showed no statistical difference between treatments LPS and MPS. It is possible that the LPS used in this study may not be “low” enough and even lower levels of predation stress need to be studied. Our low level of predation stress in this study was exposure to one predator but for all its lifespan. Two further studies of ours in this series explore reduced exposure of predation stress by reducing the duration of exposure: one exposed prey to predation stress during immature stages, oviposition period or post-oviposition (Wei, Liu and Zhang, unpublished data). Another study further reduces the duration of exposure to each immature stage, such as larva, protonymph, and deutonymph (Wei, Li and Zhang, unpublished data). The larval stage lasted only 3 or 4 days on average and this short exposure to predation stress could extend prey lifespan. Thus, different species may show different strategies to respond to predation stress, and different levels of predation stress may generate different effects on prey life history. Similar responses were seen when animals were exposed to other types of stress. For example, exposure to both extreme nonlethal chilling and heat decreased the longevities of females (Søvik and Leinaas 2003; Jiao et al. 2016; Zhang et al. 2016), but repeated exposure to transient heat, which was relatively mild, showed totally opposite results (Hercus et al. 2003; Zheng et al. 2017).

Li and Zhang (2019) showed that the effects of predation stress on prey lifespan were sex-specific: only prey (*T*. *urticae*) females had reduced lifespan when exposed predation stress from *P*. *persimilis*. They proposed that this shortened lifespan of females under predation risk might be due to a combination of higher resource requirements and greater vulnerability to predation than males. In this study, we showed that both males and females of *T*. *putrescentiae* had shortened lifespan when exposed to predation stress from *N*. *cucumeris*. The females of both *T*. *urticae* and *T*. *putrescentiae* have higher resource requirements than males, and especially during oviposition periods, but the males of *T*. *putrescentiae* are not as aggressive (personal observations) as *T*. *urticae* described by Potter et al. (1976). Thus, the difference in the behaviour between males and females may be more important in shaping sex-specific responses of prey to predation stress in these species. In this study, we observed different types of sex-specific responses: (1) there was significant difference in the comparisons of LPS and MPS groups between males and females: in males, prey individuals of the MPS group lived shorter than those of the LPS group, whereas no such difference was seen in females; (2) the females had a greater reduction in lifespan than the males under LPS.

In this study, we showed that prey developmental time increased with the increasing level of predation stress for both males and females. These results are consistent with the results of previous studies that indicated predation stress could delay prey development (Beketov and Liess 2007; Li and Zhang 2019). Likewise, we also observed no significant influences in adult body size when prey were exposed to predation stress. However, a number of previous studies suggested that the prey under predation risk should have developed faster and matured at a relatively smaller size (McPeek et al. 2001; Peckarsky et al. 2002; Altwegg 2002; Dahl and Peckarsky 2003; Griffis-Kyle and Ritchie 2007; Thaler et al. 2012; Clinchy et al. 2013). This observed reduction in immature growth rate could reflect a trade-off between soma maintenance (stronger to defend/combat and therefore more chances to mate for males) and chances for reproduction (matured faster). It has also been shown that immature males seem to be less sensitive to predation stress or food shortage compared with females (Walzer and Schausberger 2011). But as mentioned before, males of *T*. *putrescentiae* may not be as bold as males of other species, such as spider mites. Both females and males of *T*. *putrescentiae* showed no trade-offs between development time and lifespan, even under different levels of predation stress.

Moreover, *T*. *putrescentiae* had a relatively longer post-oviposition period (cf oviposition period) than *T*. *urticae*. According to Li and Zhang (2019), the chronic predation stress reduced only the oviposition period and lifespan of female *T*. *urticae*, but not the post-oviposition period. However, in our results, predation risk significantly reduced the post-oviposition period of female *T*. *putrescentiae*, and with the increasing level of predation stress, the post-oviposition was shorter. Thus, different mechanisms for reduction in lifespan were present in these two species: predation risk reduced the lifespan of *T*. *urticae* under predation stress by shortening the oviposition period, whereas it reduced the lifespan of *T*. *putrescentiae* by decreasing both the oviposition and post-oviposition period.

We also showed in this study that females of *T*. *putrescentiae* under predation stress had a reduction in fecundity, which is similar to reports in previous studies (Zanette et al. 2011; Li and Zhang 2019), and also a reduction of lifespan, which is similar to results of Li and Zhang (2019). The positive correlation between fecundity and lifespan is the opposite of a trade-off between the lifespan and reproduction in some other species, such as neriid flies under mild stress (Adler et al. 2013, 2016; Rodríguez-Muñoz et al. 2019). The results of this positive relationship between fecundity and lifespan of *T*. *putrescentiae* may demonstrate that these mites failed to balance the energy use under such strong predation stresses, which were supplied during the whole lifespan of the prey (Li and Zhang 2019). In further studies, we expose *T*. *putrescentiae* to milder stress from the predators by reducing duration of exposure to immature stages or part of the adult stages (Wei, Liu and Zhang in preparation; Wei, Li and Zhang, unpublished data).
